# ALCAP2 inhibits lung adenocarcinoma cell proliferation, migration and invasion via the ubiquitination of β-catenin by upregulating the E3 ligase NEDD4L

**DOI:** 10.1038/s41419-021-04043-6

**Published:** 2021-07-31

**Authors:** Weijie Zhang, Ruochen Zhang, Yuanyuan Zeng, Yue Li, Yikun Chen, Jieqi Zhou, Yang Zhang, Anqi Wang, Jianjie Zhu, Zeyi Liu, Zhaowei Yan, Jian-an Huang

**Affiliations:** 1 Department of Pulmonary and Critical Care Medicine, the First Affiliated Hospital of Soochow University, Suzhou, 215006 China; 2 Institute of Respiratory Diseases, Soochow University, Suzhou, 215006 China; 3 Suzhou Key Laboratory for Respiratory Diseases, Suzhou, 215006 China; 4 Department of Pharmacy, the First Affiliated Hospital of Soochow University, Suzhou, 215006 China

**Keywords:** Drug development, Non-small-cell lung cancer

## Abstract

Lung cancer is recognized as the leading cause of cancer-related death worldwide, with non-small cell lung cancer (NSCLC) being the predominant subtype, accounting for approximately 85% of lung cancer cases. Although great efforts have been made to treat lung cancer, no proven method has been found thus far. Considering β, β-dimethyl-acryl-alkannin (ALCAP2), a natural small-molecule compound isolated from the root of Lithospermum erythrorhizon. We found that lung adenocarcinoma (LUAD) cell proliferation and metastasis can be significantly inhibited after treatment with ALCAP2 in vitro, as it can induce cell apoptosis and arrest the cell cycle. ALCAP2 also significantly suppressed the volume of tumours in mice without inducing obvious toxicity in vivo. Mechanistically, we revealed that ALCAP2-treated cells can suppress the nuclear translocation of β-catenin by upregulating the E3 ligase NEDD4L, facilitating the binding of ubiquitin to β-catenin and eventually affecting the wnt-triggered transcription of genes such as survivin, cyclin D1, and MMP9. As a result, our findings suggest that targeting the oncogene β-catenin with ALCAP2 can inhibit the proliferation and metastasis of LUAD cells, and therefore, ALCAP2 may be a new drug candidate for use in LUAD therapeutics.

## Introduction

Lung cancer is a major public health problem and causes the highest number of cancer-related deaths. Non-small-cell lung cancer (NSCLC), a subtype of lung cancer, accounts for approximately 85% of newly diagnosed cases of lung cancer [1], and adenocarcinoma is the main histological type of NSCLC [2]. EGFR mutations are the most common type of mutation in lung adenocarcinoma (LUAD), and the therapeutic strategies and prognoses of patients have been greatly improved with advances in EGFR-TKIs [3]. However, the 5-year survival rate of NSCLC patients is only approximately 24% since most patients eventually develop acquired resistance to first-generation EGFR-TKIs after 12–24 months of treatment [4], and EGFR wild-type patients do not benefit from targeted therapies, in contrast to EGFR mutant patients, while KRAS-mutant patients are still in lack of target therapies [5]. Therefore, it is essential to find a therapeutic plan and develop combinational therapies to improve treatment outcomes.

*Lithospermum erythrorhizon* is a plant used in traditional Chinese medicine. It has long been used to treat burns, macules and sore throat, and its roots contain naphthoquinone [6]. Natural naphthoquinone compounds can be classified into two enantiomers, alkannin and shikonin [7]. A previous study reported that shikonin and its derivatives have many pharmacological functions, such as anti-inflammatory, antitumor [8], antioxidant [9] and immunoregulatory [10] functions. β, β-Dimethyl-acryl-alkannin (ALCAP2), one of the main active ingredients in the root of *Lithospermum erythrorhizon*, is in a class of highly fat-soluble naphthoquinone compounds [7, 11]. The antitumour roles of ALCAP2 have been reported in many carcinomas, and ALCAP2 has been shown to exert cytotoxic effects in breast cancer, leukaemia, colorectal cancer cells [11, 12], and other types of cancer cells. ALCAP2 can inhibit the activity of topoisomerase I enzyme, subsequently inducing the DNA damage response and mitochondrial apoptosis by producing reactive oxygen species in the K562 leukaemia cell line [13], but no study has been performed to detect its role in NSCLC cells.

The Wnt signalling pathway is highly conserved in cancer and contains three main branches: the Wnt/β-catenin pathway, which is also known as the canonical Wnt pathway, the noncanonical Wnt-calcium (Wnt-Ca^2+^) pathway and the Wnt–planar cell polarity (Wnt-PCP) pathway. The most well-studied pathway is the Wnt/β-catenin pathway [14]. The Wnt/β-catenin signalling pathway plays roles in different diseases. We observed abnormal activation of the Wnt/β-catenin pathway in fibrotic disorders, similar to renal fibrosis and IPF [15, 16], as the Wnt/β-catenin pathway can promote TGF-β-mediated fibroblast activation. Inappropriate activation of the Wnt/β-catenin pathway is also common in cancers; it can regulate the EMT, the cell cycle and tumour cell apoptosis, thus affecting the proliferation and metastasis of breast cancer, gastric cancer lung cancer, etc. [17–19].

Recent studies have demonstrated that β-catenin is a substrate for ubiquitination. E3 ligases, including SHPRH, β-TrCP and CBL, have been found to mediate β-catenin ubiquitination [20–22]. Neural precursor cell expresses developmentally downregulated 4-like (NEDD4L), a member of the NEDD4 family that contains a HECT domain for Ub protein ligation [23]. NEDD4L has been reported to be downregulated in multiple types of cancers and acts as a tumour suppressor gene in melanoma [24], colorectal cancer [25], lung cancer [26], and other cancers.

Our study elucidated the antitumour effects of ALCAP2 in LUAD cells and the underlying mechanisms of its action in the present study. Briefly, ALCAP2 suppresses the proliferation, migration and invasion of cells, arrests the cell cycle or promotes apoptosis by facilitating the ubiquitination of β-catenin via the upregulation of the E3 ligase NEDD4L.

## Materials and methods

### Materials

A549, H1299, HCC827, HEK 293T and BEAS-2B (human immortalized normal epithelial cells) cells were purchased from the Cell Bank of the Chinese Academy of Sciences (Shanghai, China). Cells were cultured in RPMI 1640 medium and DMEM with 10% foetal bovine serum (Gibco, Carlsbad, CA, USA) and L-glutamine (Invitrogen, Carlsbad, CA, USA) at 37 °C in a 5% CO_2_ atmosphere. ALCAP2 (purity > 98%) was purchased from the National Institutes of Food and Drug Control of China (Beijing, China).

### MTT assays

Cells were seeded into 96-well plates (3000 cells/well) in 100 μl of medium containing 10% FBS for 24 h, and fresh medium containing ALCAP2 at different concentrations (0.1–5 mmol) was incubated with cells for 24 h. The medium was removed, and the cells were cultured with MTT (0.5 mg/ml, 100 μl MTT/well) for 3–4 h. Then, the absorbance of DMSO-dissolved blue formazan crystals was read and quantitated.

### Colony formation and EdU assays

Tumour cells were treated with or without different concentrations of ALCAP2 for 24 h, and then, 3000 cells were seeded in a 60-mm plate containing complete culture medium. The cells were cultured for 7–10 days until foci formed. The cells were washed with PBS twice, fixed with methanol, and stained with crystal violet. The EdU assay kit was purchased from RiboBio Company (C10310-1, Guangzhou, China). We performed experiments strictly following the kit instructions. An Olympus IX-73 inverted microscope (Tokyo, Japan) was used to take images.

### Migration and invasion assay

For the migration assay, cells treated with or without different concentrations of ALCAP2 were plated into the upper chamber of a Transwell insert in medium containing 1% FBS. The difference between the migration assay and invasion assay is that in invasion assay, the upper chamber is precoated with a Matrigel matrix (BD Science, Sparks, MD, USA). In the upper chamber, 3 × 10^4^ cells were seeded for the migration assay, while 5 × 10^4^ cells were seeded for the invasion assay. Then, 800 μl of complete medium was added to the lower chamber. After 24 h of incubation, the cells that invaded from the upper surface to the lower chamber were fixed with methanol and stained with crystal violet. Finally, three random fields from each Transwell sample were selected to examine under a light microscope.

### Apoptosis and cell cycle analysis

Tumour cells were treated with or without different concentrations of ALCAP2 for 24 h. An apoptosis and cell cycle analysis kit was purchased from Beyotime Biotechnology (Shanghai, China). For the apoptosis assay, cells were stained with buffer containing Annexin V/FITC or APC and PI. For the cell cycle analysis, cells were suspended overnight in 70% ethanol at 4 °C and stained with a PI/RNase mixture in the dark at 37 °C for 30 min. The stained cells were detected in a FACSCalibur system (Beckman Coulter, Brea, CA, USA).

### RNA sequencing

The dosing group and control group were evaluated in a 3-to-3 ratio. After treatment with ALCAP2 at a concentration of 0.6 μM or in total medium containing 1% DMSO for 24 h, the cells were lysed with 1 ml of RNAiso Plus (TaKaRa, Osaka, Japan). RNA sequencing was performed in the Illumina HiSeqXTen sequencing platform (NovelBio Bio-Pharm Technology Co. Ltd., Shanghai).

### Protein stability assays

CHX is an inhibitor of protein synthesis, and we used it to detect the half-life of β-catenin. Cells were treated with CHX (100 μg/ml) in separate groups for 1, 2, 4, 6 and 8 h before protein extraction. β-catenin degradation was analysed by western blot analysis.

### Western blot analysis

Western blot analysis was performed as we previously described [27]. The following antibodies were used in the analysis: anti-β-catenin, anti-p-β-catenin, anti-NEDD4L, anti-Akt, anti-p-Akt (Ser473), anti-PARP, anti-Survivin, anti-MMP9, anti-CBL (Cell Signaling Technology), anti-N-cadherin, anti-E-cadherin, anti-vimentin (BD Biosciences, USA), anti-cyclin D1, anti-H2AX (Abcam, London, UK), anti-tubulin, anti-Lamin-B1 (Proteintech Group, Inc), anti-β-actin, anti-mouse and anti-rabbit secondary antibodies (Cell Signaling Technology).

### Cellular fractionation

A nuclear protein extraction kit was purchased from TransGen Biotech (DE201-01, TransGen Biotech) for use in cellular fraction experiments. Generally, cells were first lysed with cytoplasmic protein extraction buffer and then with nuclear extraction buffer to obtain the nuclear fraction.

### RNA interference

The small interfering RNA (siRNA) sequences corresponding to the target sequences were directly synthesized (GenePharma). The siRNA constructs were as follows: siRNA-NEDD4L: 5′-CAGAAATAATGGTCACAAA-3′ (sense) and 5′-TTTGTGACCATTATTTCTG-3′ (antisense). Transfection of siRNA into cells was performed with Lipofectamine 2000 according to the instructions of the manufacturer.

### Plasmid construction

The CDS region of β-catenin was amplified using cDNA from H1299 cells as the template and cloned into a PCDNA3.1-Myc plasmid. The primer sequences used for the construction of c-Myc-β-catenin are shown in Table 1.

**Table 1:** Primers sequences used in the study.

Gene	Primer Sequences
β-catenin	F: CATCTACACAGTTTGATGCTGCT R: GCAGTTTTGTCAGTTCAGGGA
NEDD4L	F: ATTTTCCACGGCCATGAGA R: TCCAATGGTCCTCAGCTGTTTA
CBL	F: TAGGCGAAACCTAACCAAACTG
	R: AGAGTCCACTTGGAAAGATTCCT
SP1	F: TGGCAGCAGTACCAATGGC
	R: CCAGGTAGTCCTGTCAGAACTT
DDB2	F: CTCCTCAATGGAGGGAACAA
	R: GTGACCACCATTCGGCTACT
β-actin	F: CACAGAGCCTCGCCTTTGC
	R: ACCCATGCCCACCATCACG
c-Myc-β-catenin	F: ATAAGAATGCGGCCGC ATGGCTACTCAAGCTGATTT
	R: GGTACCGGTCAGGTCAGTATCAAACCAGGCCA

### RNA extraction, cDNA synthesis and quantitative real-time PCR analysis

The detailed processes were performed as we previously described [28]. The primers used in the study are listed in Table 1. The CT values of the gene mRNA levels were normalized to those of β-actin, the internal control. The ^△△^Ct method was applied to calculate the relative quantities of these mRNAs. Each experiment was performed in triplicate.

### Immunofluorescence

After treatment with ALCAP2 for 24 h, cells were plated onto coverslips. The cells were fixed with 4% formaldehyde for 15 min and then permeabilized with 0.5% Triton X-100 PBS solution for 20 min at room temperature. After blocking with 5% BSA for 30 min, the cells were incubated overnight with an anti-β-catenin primary antibody at 4 °C. The cells were incubated with the corresponding secondary antibody labelled with Cy3 for 1 h in the dark. Nuclei were stained with DAPI for 5 min. Images of the stained cells were taken with a Leica SP8 confocal microscope under standardized conditions.

### Co-immunoprecipitation (co-IP) assay

Cells not treated or treated with ALCAP2 were lysed with 1 ml of RIPA buffer (Cell Signaling Technology, Danvers, MA, USA) for 30 min, and then, the cells were collected by scraping and the protein in the lysates were incubated overnight with normal rabbit IgG antibody or anti-β-catenin at 4 °C with rotation. On day 2, the mixture was incubated with protein A/G beads or anti-c-Myc magnetic beads at 4 °C for 4 h. After washing with lysis buffer three times, the beads were boiled in 2× SDS protein loading buffer and then subjected to western blot analysis.

### In vivo tumour xenograft animal model

The animal experiment was approved by the Ethics Committee of the First Affliated Hospital of Soochow University. Female BALB/c nude mice (3–4 weeks old weighing 16–20 g) were obtained from the Experimental Animal Center of Soochow University. A total of 1.5 × 10^6^ A549 cells were inoculated subcutaneously into the flanks, and the female mice were randomly divided into two groups (six mice per group): a castor oil control group and an ALCAP2 group (14 mg kg^−1^). When the tumour weight reached nearly 100 mm³, castor oil or ALCAP2 were administered to mice every 2 days via intraperitoneal injection. We evaluated tumour growth by volume (*V*), calculating with the formula *V* = *L* (tumour length) × *W* (tumour width)^2^/2.

### Statistical analysis

Differences between the two groups were assessed by unpaired Student’s *t* test. All results were presented as the means±SD. Statistical analyses were performed using GraphPad Prism 8 software (GraphPad, San Diego, CA, USA). Differences for which *P* < 0.05 were considered significant.

## Results

### ALCAP2 inhibits the proliferation of LUAD cells in vitro

**Fig. 1:**
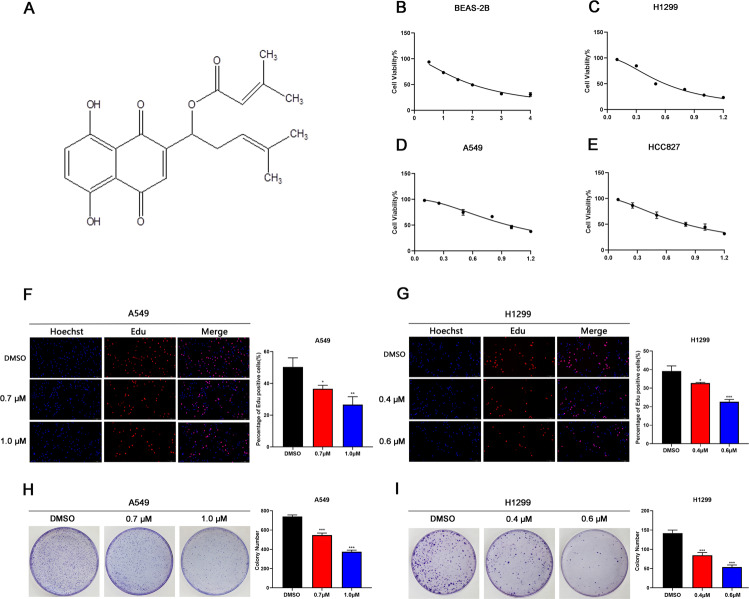
ALCAP2 inhibits the proliferation of LUAD cells. **A** Chemical structure of ALCAP2. **B**–**E** The viability of A549, H1299, and HCC827 cells and bronchial epithelial BEAS-2B cells was determined by MTT assay after the cells treated with ALCAP2 at the indicated concentrations. The IC50 of ALCAP2 for each cell line was calculated according to a cell viability value. **F**, **G** An EdU staining assay was performed to determine the proliferation capacity of A549 and H1299 cells treated with or without ALCAP2. EdU-positive ratios are shown. Scale bar, 2 mm. **H**, **I** Representative images of the results of the clonogenic analysis of A549 and H1299 cell proliferation after treatment with or without ALCAP2. Student’s *t* test was used for statistical analysis and data were presented as the mean ± SD. *P < 0.05; **P < 0.01; ***P < 0.001.

LUAD cells possess a high capacity for proliferation, migration and invasion. To find a new medicine that can inhibit LUAD cell proliferation, we investigated the effect of ALCAP2. The Chemical structure of ALCAP2 was shown in Fig. 1A. The MTT assay results showed that incubation with ALCAP2 for 24 h led to substantial declines in cell viability, the half-maximal inhibitory concentration (IC50) of the ALCAP2 effect on cell viability was calculated to be 0.72 μM for HCC827 cells, 0.96 μM for A549 cells and 0.59 μM for H1299 cells (Fig. 1C–E), we found that ALCAP2 did not kill bronchial epithelial BEAS-2B cells with the effectiveness as NSCLC cells, as the IC50 of ALCAP2 in BEAS-2B cells was 2.01 μM (Fig. 1B). We used the EdU assay to detect the number of cells that were in the proliferative phase. We found that the number of ALCAP2-treated cells in the proliferative phase were reduced compared with the number of cells not treated with ALCAP2 (Fig. 1F, G). We also confirmed these results by colony formation assays (Fig. 1H, I).

### ALCAP2 arrests the cell cycle and promotes LUAD cells apoptosis

**Fig. 2:**
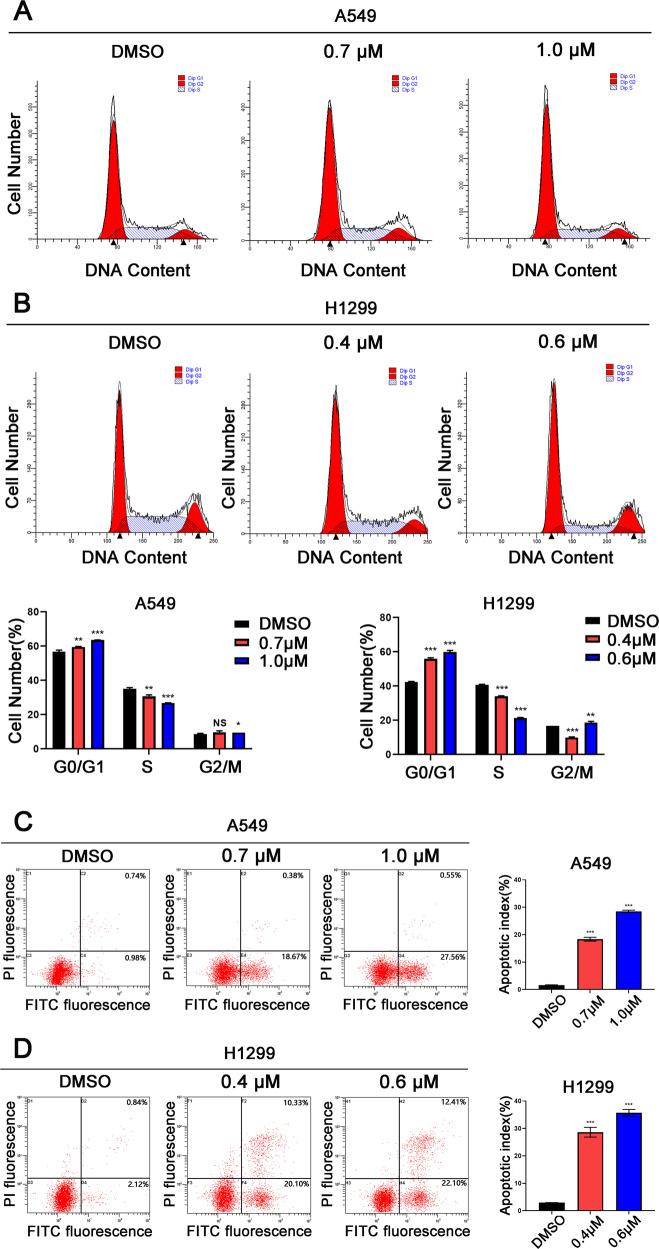
ALCAP2 arrests the cell cycle and promotes the apoptosis of LUAD cells. **A**, **B** Flow cytometry assay of LUAD cell lines. The cells were harvested at 24 h after treatment with or without ALCAP2 and stained with PI. The percentage of cells in each cell cycle phase is shown in the inset of each panel. **C**, **D** Flow cytometry assay of A549 and H1299 cells treated with or without ALCAP2. Cells harvested at 24 h after transfection and stained with Annexin V-FITC and PI. The right histogram panel shows the statistics of the number of apoptotic cells in each group. The data were presented as the mean ± SD of three measurements. NS: no significance, *P < 0.05; **P < 0.01; ***P < 0.001.

Flow cytometry assays were performed to detect changes in the cell cycle and apoptosis. We found that the proportion of ALCAP2-treated cells in the G0/G1 phase was increased and that the proportion of these cells in the S phase was decreased compared with the cells not treated with ALCAP2 (Fig. 2A, B). The flow cytometry results also showed an increase in the ALCAP2-treated cells undergoing apoptosis (Fig. 2C, D). Treatment with ALCAP2 led to either a change in the cell cycle or apoptosis, with both effects trending toward increasing significance with increasing dose concentration. These results suggested that ALCAP2 can inhibit the proliferation of LUAD cells by affecting the cell cycle and apoptosis.

### ALCAP2 suppresses the migration and invasion of LUAD cells

**Fig. 3:**
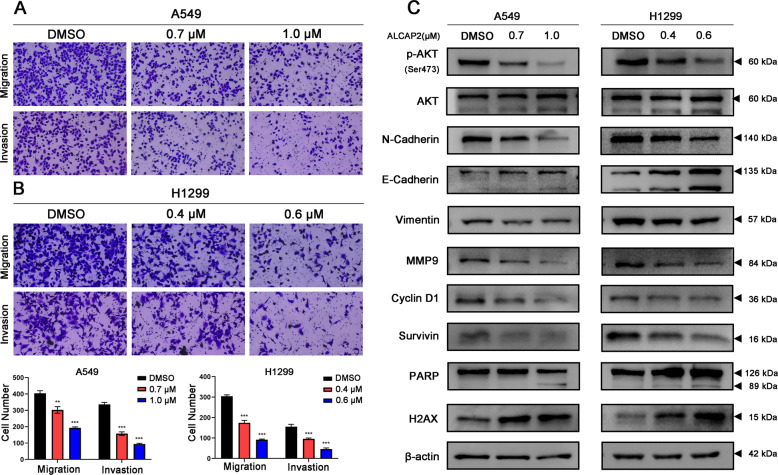
ALCAP2 suppresses the migration and invasion of LUAD cells. **A**, **B** Representative images showing the results of Transwell cell migration and invasion assays of A549 and H1299 cells treated with or without ALCAP2. **C** The protein expression of N-cadherin, E-cadherin, vimentin, MMP9, Akt, p-Akt, H2AX, cyclin D1, PARP and survivin in A549 and H1299 cells treated with or without ALCAP2 as determined by western blot analysis. The data were analysed by unpaired Student’s t-test, **P < 0.01, and ***P <0.001 vs the control.

Transwell assays were performed to detect the effects of ALCAP2 on LUAD cells migration and invasion. After treatment with ALCAP2, dose-dependent inhibition of migration and invasion was evident for the A549 and H1299 cells (Fig. 3A, B).

Next, we performed western blotting to explore the potential mechanism by which ALCAP2 inhibits metastasis and proliferation. We found that the expression of the EMT-related markers N-cadherin and vimentin and the matrix metalloproteinase MMP9 was significantly decreased after cells were treated with ALCAP2 and that the level of E-cadherin was increased. Additionally, the protein levels of p-Akt, cyclin D1 and survivin were decreased, while those of PARP and H2AX were increased (Fig. 3C). These results indicated that ALCAP2 can suppress the migration and invasion of LUAD cells.

### ALCAP2 can suppress the protein level of β-catenin

**Fig. 4:**
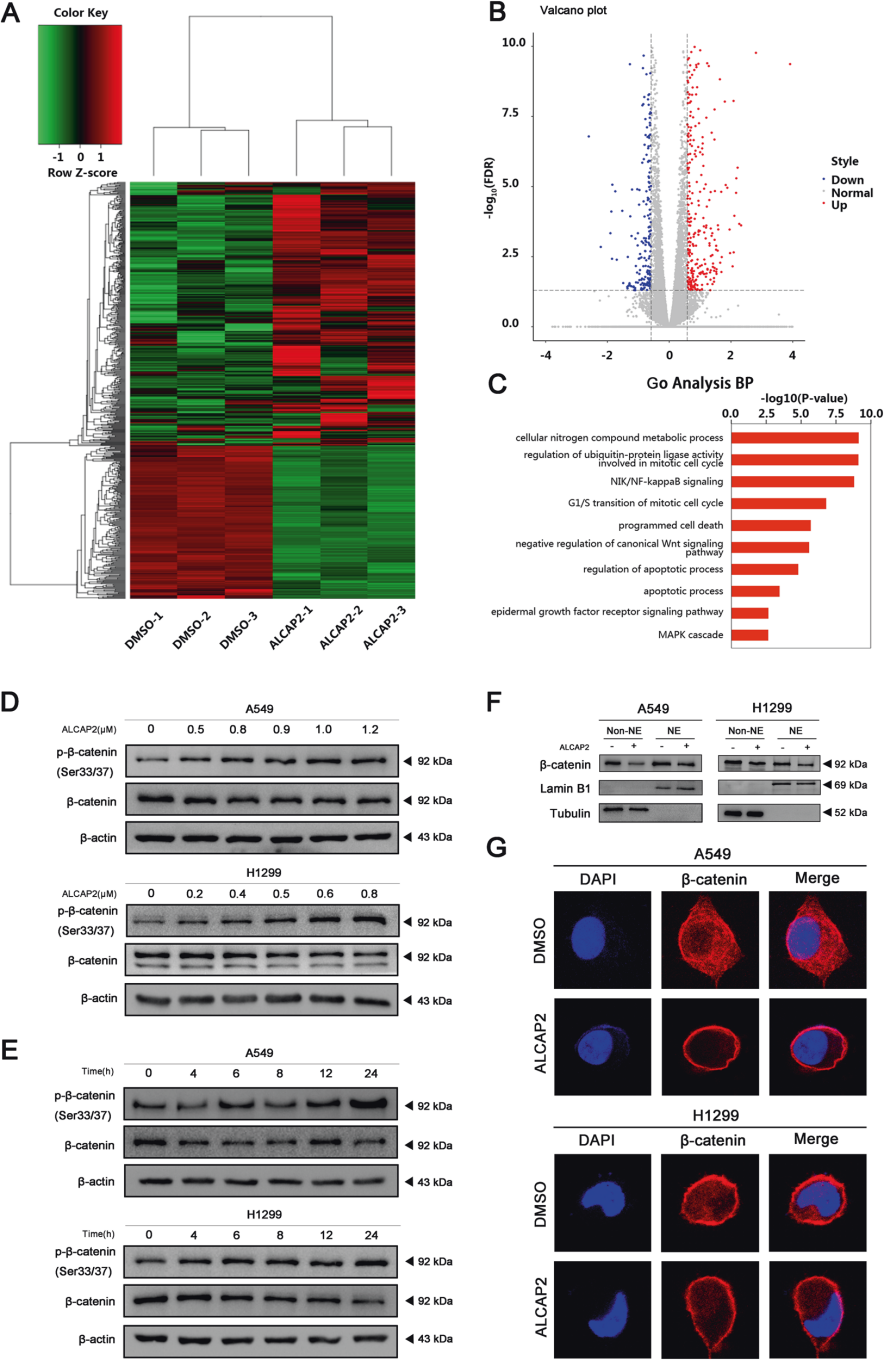
ALCAP2 suppresses the total and nuclear protein levels of β-catenin. **A**–**C** Heat map of differentially expressed mRNAs in the treatment and control groups. A total of 592 genes were identified [false discovery rate (FDR) <0.05 and absolute fold change (FC absolute) >0.585], among which there were 373 were upregulated and 219 were downregulated. Then, we performed a GO enrichment analysis of these genes. **D** A549 and H1299 cells were treated with different concentration of ALCAP2 for 24 h, cells were lysed with 1 ml of RIPA buffer for western blot. **E** A549 were treated with 1.0 μM ALCAP2 and H1299 treated with 0.6 μM ALCAP2 for different time, then cells were lysed with 1 ml of RIPA buffer for western blot. **F** A549 and H1299 cells treated with or without ALCAP2 were subjected to cellular fractionation followed by western blotting for detecting β-catenin. Tubulin and Lamin B1 are endogenous markers of cytosolic and nuclear proteins (NE: Nuclear, Non-NE: Cytoplasmic). **G** Immunofluorescence staining of β-catenin after A549 and H1299 cells were treated with or without ALCAP2.

Next, to determine how ALCAP2 exerts its anti-NSCLC function, RNA-Seq was performed to identify the transcriptional profile in H1299 cells after treatment with ALCAP2. We analysed the results with bioinformatics methods. In total, 592 genes were identified [false discovery rate (FDR) <0.05 and the absolute fold change (FC absolute) >0.585], among which 373 genes were upregulated and 219 genes were downregulated (Fig. 4A, B). Then, we performed GO enrichment analysis and KEGG pathway enrichment analysis (Fig. S2A) of these genes. The GO enrichment analysis results indicated that ALCAP2 negatively regulated the canonical Wnt signalling pathway (Fig. 4C). As the Wnt/β-catenin pathway plays an important role in LUAD, we sought to determine the expression of β-catenin after cells were treated with ALCAP2. The western blot analysis results showed that ALCAP2 inhibited β-catenin expression in both A549 and H1299 cells in dose and time-dependent manner, while we can obtain that the protein level of p-β-catenin, the active form of β-catenin can be promoted in dose and time-dependent manner after cells treated with ALCAP2(Fig. 4D, E).

As β-catenin is translocated to the nucleus and interacts with TCF/LEF to enhance transcription, we verified the expression of β-catenin by separating the cytoplasmic (non-NE) and nuclear (NE) fractions of ALCAP2-treated cells and control cells. We noticed that β-catenin was decreased in both the cytoplasmic and nuclear fractions (Fig. 4F). We reconfirmed the results through immunofluorescence analysis (Fig. 4G). While we can also obtain that there was an obvious promotion of p-β-catenin in cytoplasm, while the level of p-β-catenin in nuclear was upregulated too, but it is not as obvious as it in cytoplasm (Fig. S3A).

### ALCAP2 inhibits the expression of β-catenin by facilitating its ubiquitination

**Fig. 5:**
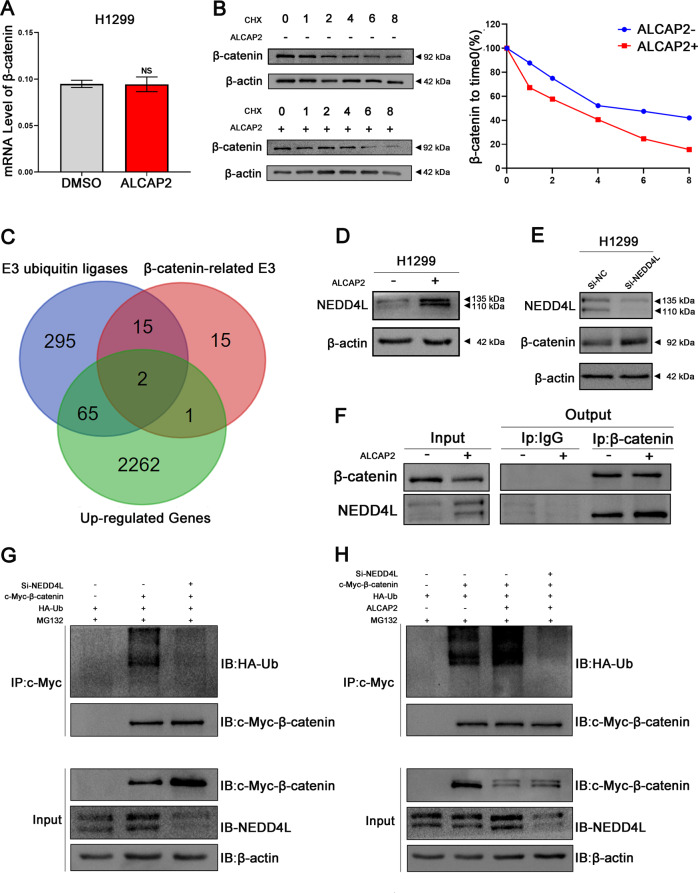
ALCAP2 promotes β-catenin ubiquitination by increasing the expression of NEDD4L. **A** The mRNA level of β-catenin was not significantly different after treatment with ALCAP2. **B** The degradation of β-catenin mediated by CHX. H1299 cells treated with or without ALCAP2 were seeded into six-well plates. After 24 h, the cells were treated with 100 µg/ml CHX. Cell lysates were prepared for western blot analysis. **C** Three databases include upregulated genes in the RNA-Seq results [absolute fold change (FC absolute) >0.1 and *p* < 0.05], definitive human E3 ligases and E3 ligases related to β-catenin via UbiBrowser, were used to predict the E3 ligases of β-catenin. **D** Protein levels of NEDD4L were increased after H1299 cells were treated with ALCAP2. **E** NEDD4L downregulation increased β-catenin protein expression. **F** The co-immunoprecipitation assay proved the association between β-catenin and NEDD4L. **G** The co-immunoprecipitation assay proved that NEDD4L can promote the binding of β-catenin and Ub. **H** Co-immunoprecipitation of β-catenin and Ub showed that ALCAP2 promoted the binding of β-catenin and Ub, and this effect was reversed in si-NEDD4L-expressing cells.

Our RNA-Seq results revealed that the mRNA expression change of β-catenin after cell treatment with ALCAP2 was not significant (Fig. S1A). We performed the same experiment with H1299 cells and obtained the same results (Fig. 5A). As β-catenin can be degraded by ubiquitin, we hypothesized that ALCAP2 attenuated β-catenin protein levels through proteasomal degradation. Protein stability assays were performed with H1299 cells to determine whether ALCAP2 can affect the half-life period of β-catenin. The results showed that compared with cells not treated with ALCAP2, β-catenin was degraded more quickly after treatment with ALCAP2 (Fig. 5B). E3 ligases often determine the specificity of ubiquitin-proteasome protein degradation; therefore, we identified genes from 3 databases that were upregulated genes in RNA-Seq results [absolute fold change [FC absolute) >0.1 and p < 0.05], were identified as human E3 ligases related to β-catenin, as determined via UbiBrowser (http://ubibrowser.ncpsb.org/ubibrowser/), which led to the identification of 2 E3 ligases (Fig. 5C and Fig S1B, C). We verified the results in H1299 cells, finding that treatment with ALCAP2 increased the expression of NEDD4L at both the mRNA and protein level (Fig. S1D, E and Fig. 5D). Of these two candidates, we choose NEDD4L as the E3 ligase of β-catenin to study as we did not find an obvious raise of β-catenin after transfected with CBL si-RNA (Fig. S3B).

Next, we verified the regulatory effect of NEDD4L on β-catenin. NEDD4L downregulation increased β-catenin protein expression (Fig. S1F and Fig. 5E). Then, co-immunoprecipitation was performed, and we found that ALCAP2 facilitated the interaction between NEDD4L and β-catenin (Fig. 5F). Then we transfected HA-Ub and c-Myc-β-catenin plasmid to 293T cells, the co-immunoprecipitation assay proved that NEDD4L can promote the binding of β-catenin and Ub (Fig. 5G).

**Fig. 6:**
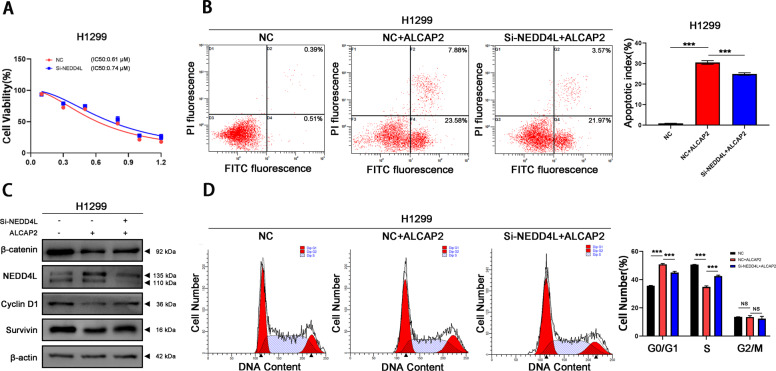
The effects of ALCAP2 on LUAD cells could be reversed by knockdown of NEDD4L. **A** The viability of si-NEDD4L H1299 cells after treated with ALCAP2 was determined by MTT assay. **B**, **D** Flow cytometry assay to detect the cell cycle and apoptosis of H1299 cells and si-NEDD4L H1299 cells treated with ALCAP2. **C** The protein expression of β-catenin, NEDD4L, cyclin D1 and survivin. The data were presented as the mean ± SD of three measurements. NS no significance, ***P < 0.001.

We examined the IC50 of ALCAP2 for 293T cells (Fig. S2B), then we identified complexes formed by β-catenin and Ub, and the results indicated that ALCAP2 distinctly increased β-catenin protein ubiquitination compared with the control, and this effect could be reversed by the expression of si-NEDD4L in cells (Fig. 5H). Then we performed MTT assay and flow cytometry assays, we found that the effects of ALCAP2 on H1299 cells can be reversed after the knockdown of NEDD4L (Fig. 6A, B, D), the western blotting results showed that the protein expressions of β-catenin, NEDD4L, cyclin D1 and survivin were also reversed too (Fig. 6C).

### ALCAP2 attenuates tumour growth in a murine xenograft model

**Fig. 7:**
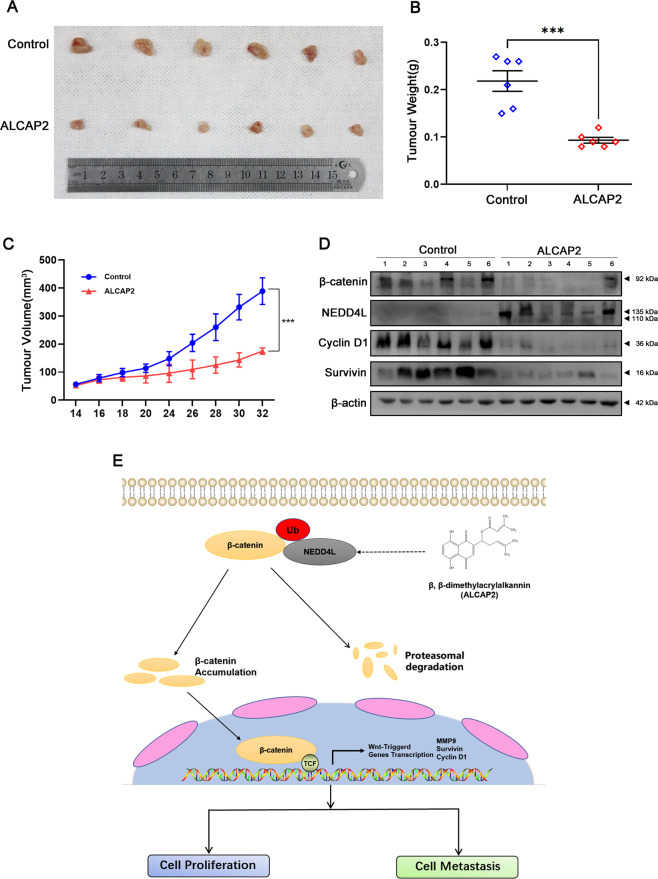
ALCAP2 attenuates tumour growth in a murine xenograft model. **A** A549 cell xenografts in nude mice (*n* = 6) at the experimental endpoint. ALCAP2 at a concentration of 14 mg/g was injected intraperitoneally every two days, and tumours were harvested and photographed as shown. **B** Tumour growth curves in mice (*n* = 6 in each group) inoculated with the indicated cells on the indicated days. **C** Each tumour was weighed. **D** β-catenin, NEDD4L cyclin D1, and survivin protein expression in tumours was measured by western blot analysis. **E** The hypothetical model showing the mechanism by which ALCAP2 plays a role in LUAD cells. ***P < 0.001.

To further assess the effect of ALCAP2 in vivo, A549 cells were injected into BALB/c athymic mice. When the tumour volume reached 100 mm^3^, ALCAP2 was injected intraperitoneally every two days at a concentration of 14 mg/g. Compared with that in the control group, the increase in tumour volume and weight was inhibited in the ALCAP2 treatment group (Fig. 7A-C). After 7 treatments, we sacrificed the mice and removed the xenograft tumours. Western blotting was performed to evaluate the protein expression of β-catenin, NEDD4L, cyclin D1 and survivin. The data indicated that ALCAP2 decreased the protein levels of β-catenin and cyclin D1 while increasing the protein level of survivin (Fig. 7D). These results were consistent with the in vitro results and confirmed that ALCAP2 can inhibit tumour growth by attenuating the expression of β-catenin in vivo.

Taken together, these data indicate that ALCAP2 inhibits LUAD cell proliferation, migration and invasion mainly by reducing the expression of β-catenin through ubiquitination induced via the upregulation of NEDD4L.

## Discussion

The antitumour role of ALCAP2 has been reported in many carcinomas, and it has been shown that ALCAP2 has cytotoxic effects in breast cancer, leukaemia and colorectal cells [11, 12], among other cells. Only one study has investigated the mechanism by which ALCAP2 inhibits cell proliferation, and this study examined K562 leukaemia cells [13]. Our study was the first to investigate the antineoplastic role of ALCAP2 in LUAD cells. We confirmed that ALCAP2 can suppress the proliferation, migration and invasion abilities of LUAD cells by arresting the cell cycle and inducing apoptosis. According to the RNA-Seq results, the underlying mechanism by which ALCAP2 affects the cell cycle and apoptosis involves the Wnt/β-catenin pathway. ALCAP2 can promote the binding of ubiquitin and β-catenin by upregulating the expression of NEDD4L, a specific E3 ubiquitin ligase of β-catenin, which leads to the nuclear translocation of β-catenin and the decreased transcription of survivin, cyclin D1 and MMP9, eventually inhibiting the ability of LUAD cells to proliferate and metastasize.

When cancer occurs, the Wnt pathway is activated, triggering the phosphorylation of Dvl, and then, the activity of GSK-3β is inhibited, thereby attenuating β-catenin phosphorylation [29] and ultimately suppressing the degradation of β-catenin and thus promoting its nuclear translocation. Through our research, we detected that ALCAP2 only can upregulate NEDD4L to promote β-catenin ubiquitination. We did not examine whether ALCAP2 can affect the expression of other genes in the complex that mediated the degradation of β-catenin, or the phosphorylation of Dvl and GSK-3β.

An increasing number of studies have shown that the Wnt/β-catenin pathway plays roles in different cancers, and we recapitulated the inappropriate activation of this pathway in gastric cancer, leukaemia and nasopharyngeal carcinoma [30–32], Wnt/β-catenin alterations are prominent in NSCLC, and lncRNA FEZF1-AS1 can facilitate the proliferation, migration and invasion of NSCLC cells by supressing the expression of AXIN1 to decrease the degradation of β-catenin [33]. The Wnt/β-catenin pathway is also proposed as a potential therapeutic target in lung cancer, and a variety of Wnt/β-catenin pathway inhibitors have shown good efficacy in cancer treatment. Stem cells are defined as having the ability to self-renew and the ability to differentiate into cells of specific lineages [34]. Stem-like cells have recently been reported in autochthonous mouse tumour models and tumour transplants [34–36]. DKK-1 (a Wnt antagonist) suppresses the proliferation and sphere formation of primary LUAD cells [37]. A study found that XAV939, a ponent tankyrase inhibitor that targets Wnt/β-catenin signalling, in combination with EGFR-TKIs can strongly inhibit β-catenin signalling and decrease the phosphorylation of EGFR compared with the effects of EGFR-TKIs alone in NSCLC cells harbouring a mutation in codon 790 (T790M) [38]. Many lines of evidence show that the Wnt/β-catenin pathway has many strong impacts on different kinds of diseases, providing a new strategie for the diagnosis and treatments of different diseases. Whether ALCAP2 can reverse cellular resistance to EGFR-TKIs remains to be determined.

We found that ALCAP2 can affect β-catenin ubiquitination. The binding of ubiquitin (8.5 kDa) to a substrate protein required the cooperation of three distinct enzymes functioning in three steps [39]. The E3 ligases determines the specificity of ubiquitin-proteasome protein degradation; therefore, they have been considered to be the most important components in this ubiquitin-driven degradation machinery [40]. NEDD4L was first reported as the specific E3 ligase of β-catenin, and it can be upregulated by ALCAP2, facilitating the binding of β-catenin and Ub and eventually leading to the degradation of β-catenin. It has also been reported that NEDD4L can negatively regulate the Wnt signalling pathway by targeting Dvl2 [41] or LGR5 [42], which indicates that NEDD4L has a strong correlation with the Wnt signalling pathway. NEDD4L was downregulated in NSCLCs, and this downregulation correlated with lymph node invasion, advanced cancer stage and poor survival [26]. This evidence indicated that NEDD4L plays a role in the initiation and progression of lung cancer. This evidence leads us to speculate that identifying a combination NEDD4L and Wnt signalling pathway-related genes may be a useful strategy for improving NSCLC diagnoses.

Ubiquitination usually involves adding ubiquitin chains to target protein molecules. There may be eight different types of ubiquitin chains, of which seven involve the sequential addition of ubiquitin proteins to a lysine (K) residue of targeted protein, with one ubiquitin molecule added to the carboxy-terminal diglycine of another ubiquitin molecule. Residues subjected to polyubiquitination include K6, K11, K27, K29, K33, K48, and K63, with K48 and k63 having been the most studied [43]. K48 primarily plays roles in protein degradation and the regulation of protein stability [44]. Modification of K63 mainly affects signal transduction, DNA repair, and the regulation of protein activity [45]. However, the ubiquitinated lysine (K) residues of β-catenin that are affected by ALCAP2 remain unknown.

NEDD4L played an important role in our study, to find the mechanism responsible for upregulation of NEDD4L, we found that SP1 can directly bind to the promoter region of NEDD4L to regulate the transcription of NEDD4L [46], we knockdown SP1 with si-RNA in H1299 cells (Fig. S4A) to verify the regulatory relationships between SP1 and NEDD4L, we can found that SP1 can upregulate NEDD4L (Fig. S4B, C), but the expression of SP1 was no significant after cells treated with ALCAP2 (Fig. S4D). We shifted our attention to the other gene-DDB2, DDB2 can bind to the promoter region of NEDD4L and recruit enhancer of zeste homolog2 histone methyltransferase to repress NEDD4L transcription by enhancing histone H3 lysine 27 trimethylation (H3K27me3) at the NEDD4L promoter [47]. We can obtain that the expression of NEDD4L can be promoted after the knockdown of DDB2 (Fig. S4E, F, G), while the mRNA level of DDB2 was suppressed after cells treated with ALCAP2 (Fig. S4H), we thought that DDB2 maybe the key role that regulated the NEDD4L in our study,

In conclusion, we identified the antitumour functions of ALCAP2 in LUAD cells: arresting the cell cycle and promoting apoptosis. Furthermore, we confirmed the mechanism by which ALCAP2 modulates the cell cycle and apoptosis process: ubiquitination of β-catenin promoted via NEDD4L reduces the intracellular level of β-catenin, and the transcription of survivin, cyclin D1 and MMP9 and ultimately inhibits the proliferation, migration and invasion of LUAD. These distinctive features suggest potential roles for ALCAP2 as an anticancer drug for LUAD patients.

## Supplementary information

Figure legend of supplementary figures

Fig S1

Fig S2

Fig S3

Fig S4

Primers used in the research
