# Supplementary material for: ALCAP2 inhibits lung adenocarcinoma cell proliferation, migration and invasion via the ubiquitination of β-catenin by upregulating the E3 ligase NEDD4L
Source: Cell Death Dis. 2021 Jul 31;12(8):755. doi: 10.1038/s41419-021-04043-6 (PMC8324825; doi:10.1038/s41419-021-04043-6)
Supplement: Supplementary file 1 — Figure legend of supplementary figures [file 41419_2021_4043_MOESM1_ESM.docx]

**Fig. S1** (A-C) The mRNA levels of β-catenin, NEDD4L and CBL in RNA-seq. (D, E) The mRNA levels of CBL and NEDD4L after cells treated with ALCAP2 in H1299 cells. (F) NEDD4L mRNA levels in NEDD4L-knockdown H1299 cells. NS: no significance, **P* < 0.05; ***P* < 0.001; ****P* < 0.001.

**Fig. S2** (A) KEGG pathway enrichment. (B) The IC50 value of ALCAP2 for 293T cells.

**Fig. S3** (A) A549 and H1299 cells treated with or without ALCAP2 were subjected to cellular fractionation followed by western blotting for detecting p-β-catenin. (B) The protein level of β-catenin in CBL-knockdown H1299 cells.

**Fig. S4** (A) The knockdown efficiency of si-SP1 in H1299 cells. (B, C) The mRNA and protein level of NEDD4L after knockdown of SP1. (D) mRNA level of SP1 after cells treated with ALCAP2. (E) The knockdown efficiency of si-DDB2 in H1299 cells. (F, G) The mRNA and protein level of NEDD4L after knockdown of DDB2. (H) mRNA level of DDB2 after cells treated with ALCAP2. NS: no significance, **P* < 0.05; ***P* < 0.001; ****P* < 0.001.
