# Supplementary figures and images for: ALCAP2 inhibits lung adenocarcinoma cell proliferation, migration and invasion via the ubiquitination of β-catenin by upregulating the E3 ligase NEDD4L
Source: Cell Death Dis. 2021 Jul 31;12(8):755. doi: 10.1038/s41419-021-04043-6 (PMC8324825; doi:10.1038/s41419-021-04043-6)

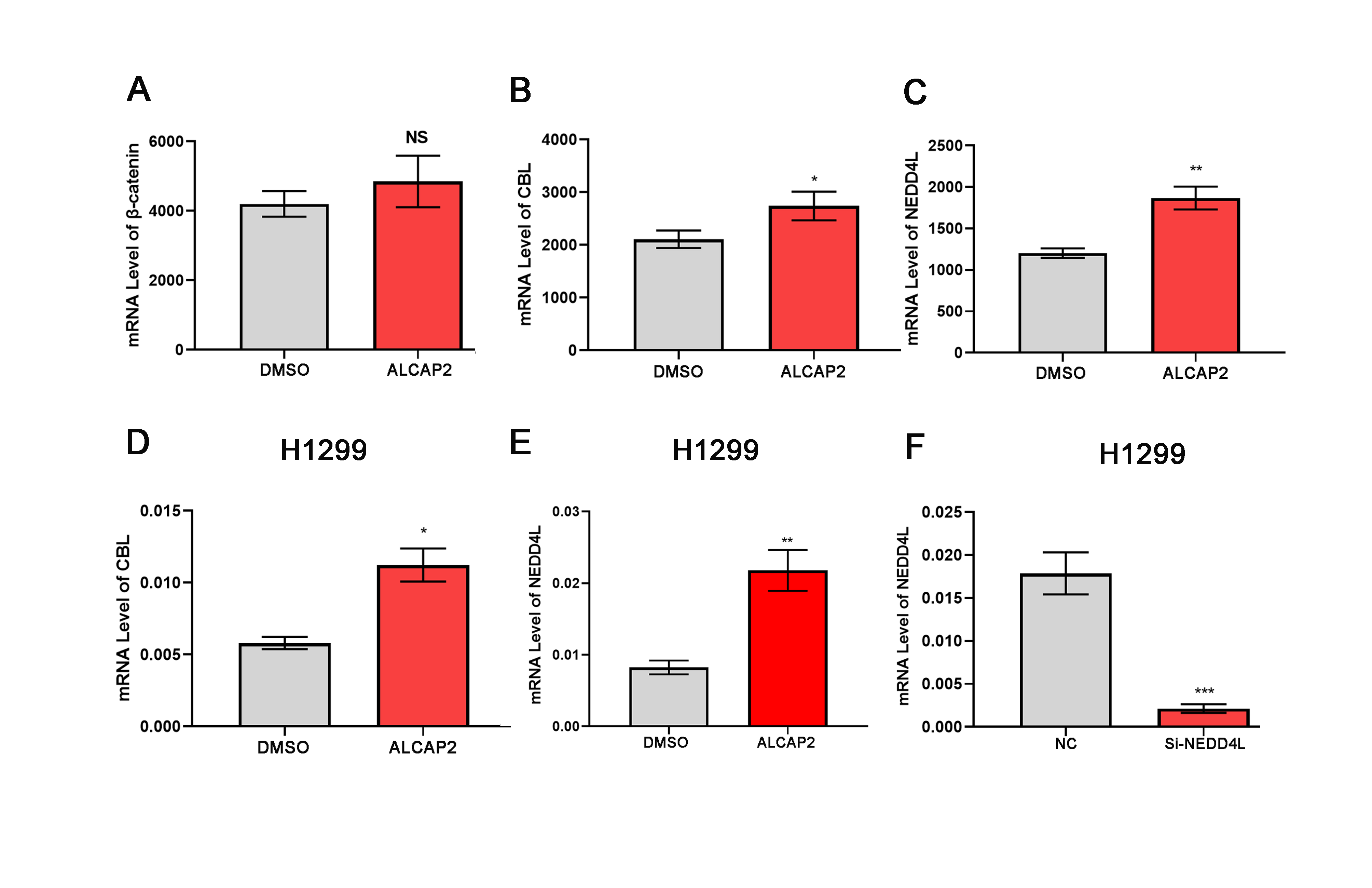

Supplement: Supplementary file 2 — Fig S1 [file 41419_2021_4043_MOESM2_ESM.tif]

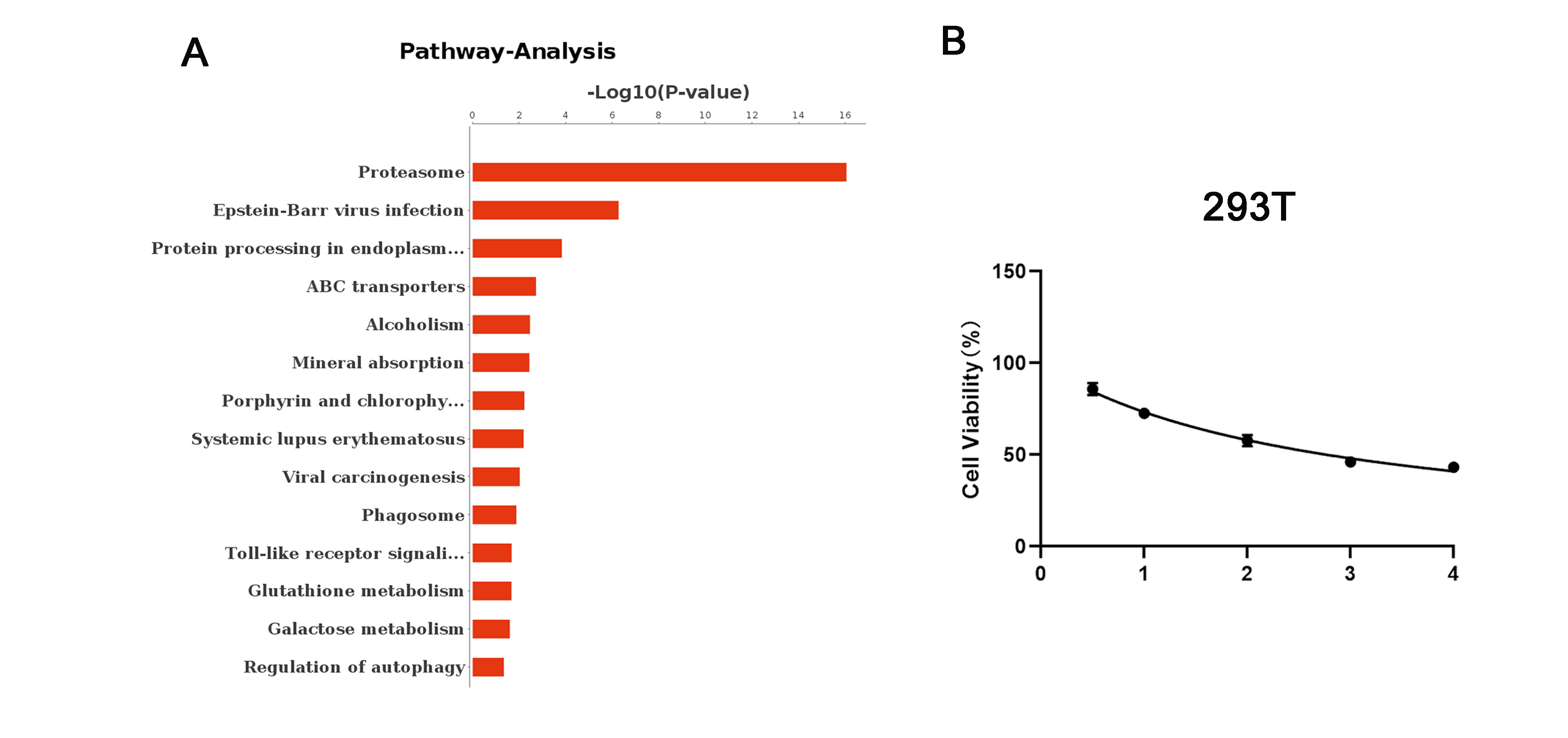

Supplement: Supplementary file 3 — Fig S2 [file 41419_2021_4043_MOESM3_ESM.tif]

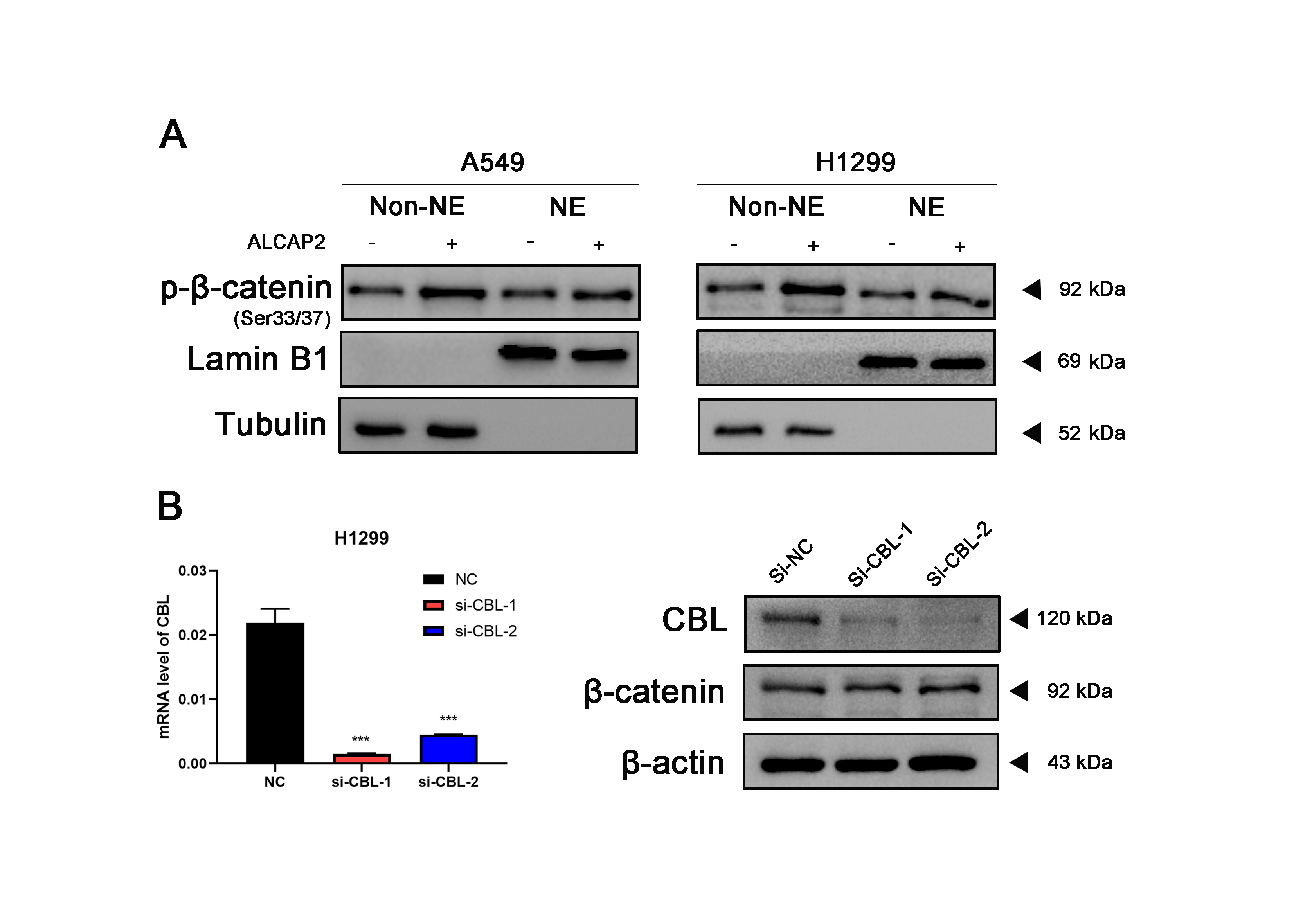

Supplement: Supplementary file 4 — Fig S3 [file 41419_2021_4043_MOESM4_ESM.tif]

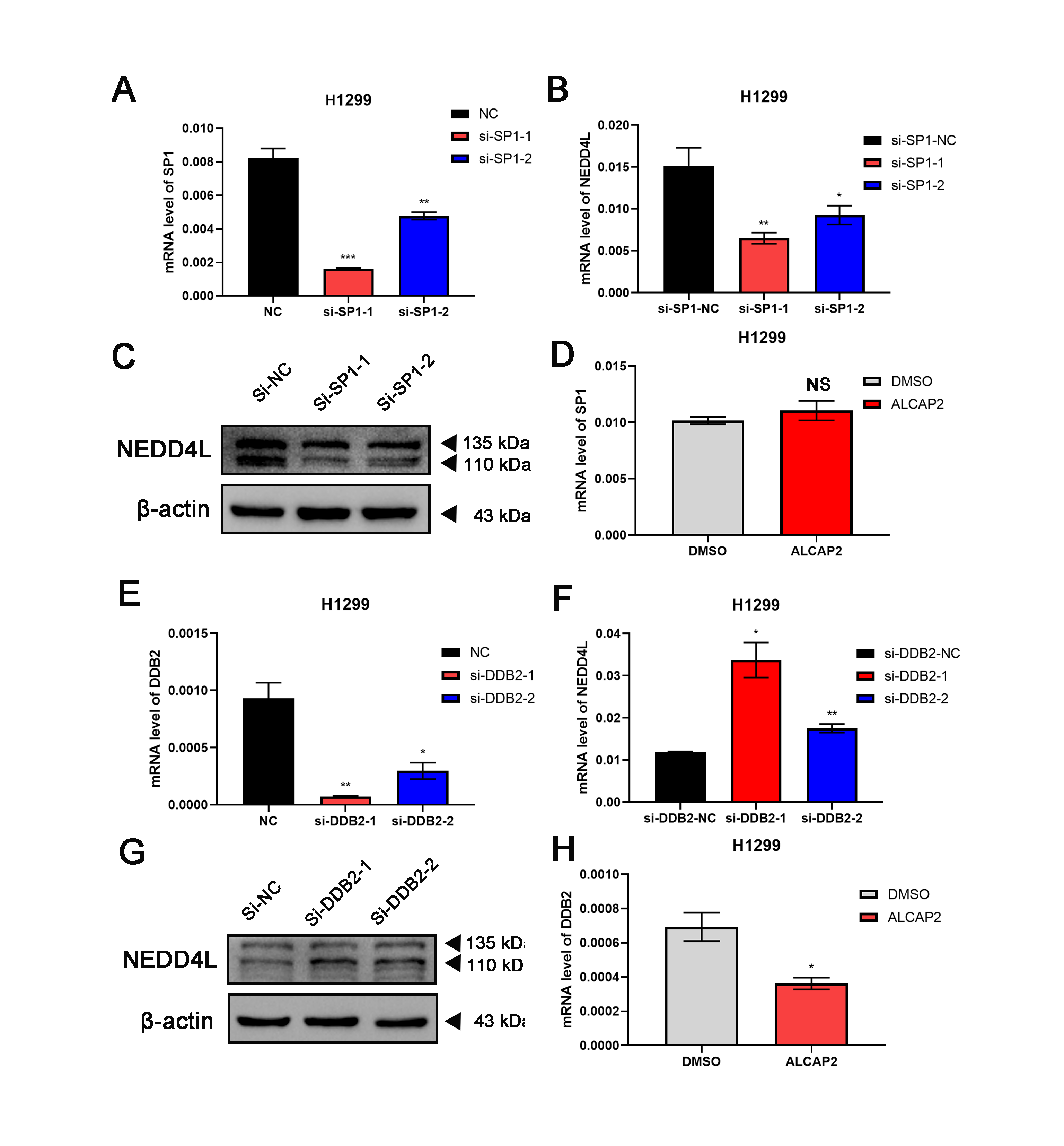

Supplement: Supplementary file 5 — Fig S4 [file 41419_2021_4043_MOESM5_ESM.tif]
