# Supplementary material for: ALCAP2 inhibits lung adenocarcinoma cell proliferation, migration and invasion via the ubiquitination of β-catenin by upregulating the E3 ligase NEDD4L
Source: Cell Death Dis. 2021 Jul 31;12(8):755. doi: 10.1038/s41419-021-04043-6 (PMC8324825; doi:10.1038/s41419-021-04043-6)
Supplement: Supplementary file 6 — Primers used in the research [file 41419_2021_4043_MOESM6_ESM.docx]

**Supplementary Table1 Primers sequences used in the study**

| **Gene** | **Primer Sequences** |
| --- | --- |
| β-catenin | F: CATCTACACAGTTTGATGCTGCT  R: GCAGTTTTGTCAGTTCAGGGA |
| NEDD4L | F: ATTTTCCACGGCCATGAGA  R: TCCAATGGTCCTCAGCTGTTTA |
| CBL | F: TAGGCGAAACCTAACCAAACTG |
|  | R: AGAGTCCACTTGGAAAGATTCCT |
| SP1 | F: TGGCAGCAGTACCAATGGC |
|  | R: CCAGGTAGTCCTGTCAGAACTT |
| DDB2 | F: CTCCTCAATGGAGGGAACAA |
|  | R: GTGACCACCATTCGGCTACT |
| β-actin | F: CACAGAGCCTCGCCTTTGC |
|  | R: ACCCATGCCCACCATCACG |
| c-Myc-β-catenin | F: ATAAGAATGCGGCCGC ATGGCTACTCAAGCTGATTT |
|  | R: GGTACCGGTCAGGTCAGTATCAAACCAGGCCA |
